# The relevancy of massive health education in the Brazilian prison system: The course “health care for people deprived of freedom” and its impacts

**DOI:** 10.3389/fpubh.2022.935389

**Published:** 2022-08-10

**Authors:** Janaína L. R. S. Valentim, Sara Dias-Trindade, Eloiza S. G. Oliveira, José A. M. Moreira, Felipe Fernandes, Manoel H. Romão, Philippi S. G. Morais, Alexandre R. Caitano, Aline P. Dias, Carlos A. P. Oliveira, Karilany D. Coutinho, Ricardo B. Ceccim, Ricardo A. M. Valentim

**Affiliations:** ^1^Laboratory of Technological Innovation in Health (LAIS), Federal University of Rio Grande do Norte (UFRN), Natal, Rio Grande do Norte, Brazil; ^2^Centre for Interdisciplinary Studies, University of Coimbra, Coimbra, Portugal; ^3^Centre for Interdisciplinary Studies, Faculty of Arts and Humanities, University of Coimbra, Coimbra, Portugal; ^4^Institute of Human Formation with Technologies, State University of Rio de Janeiro (UERJ), Rio de Janeiro, Paraná, Brazil; ^5^Department of Education and Distance Learning (DEED), Open University (Universidade Aberta), Lisbon, Portugal; ^6^International Council for Open and Distance Education, Oslo, Norway; ^7^Postgraduate Program in Education, Federal University of Rio Grande do Sul (UFRGS), Porto Alegre, Rio Grande do Sul, Brazil

**Keywords:** prison health, prison system, Brazilian prison system, public health, health education, massive health education, situated learning, massive education

## Abstract

**Introduction:**

Brazil has one of the largest prison populations globally, with over 682,000 imprisoned people. Prison health is a public health emergency as it presents increasingly aggravating disease rates, mainly sexually transmitted infections (STI). And this problem already affects both developed and developing nations. Therefore, when thinking about intervention strategies to improve this scenario in Brazil, the course “Health Care for People Deprived of Freedom” (ASPPL), aimed at prison health, was developed. This course was implemented in the Virtual Learning Environment of the Brazilian Health System (AVASUS). Given this context, this study analyzed the aspects associated with massive training through technological mediation and its impacts on prison health.

**Methods:**

This cross-sectional study analyzed data from 8,118 ASPPL course participants. The data analyzed were collected from six sources, namely: (i) AVASUS, (ii) National Registry of Health Care Facilities (CNES), (iii) Brazilian Occupational Classification (CBO), (iv) National Prison Department (DEPEN); (v) Brazilian Institute of Geography and Statistics (IBGE); and the (iv) Brazilian Ministry of Health (MoH), through the Outpatient Information System of the Brazilian National Health System (SIA/SUS). A data processing pipeline was conducted using Python 3.8.9.

**Results:**

The ASPPL course had 8,118 participants distributed across the five Brazilian regions. The analysis of course evaluation by participants who completed it shows that 5,190 (63.93%) reported a significant level of satisfaction (arithmetic mean = 4.9, median = 5, and standard deviation = 0.35). The analysis revealed that 3,272 participants (40.31%) are health workers operating in distinct levels of care. The prison system epidemiological data shows an increase in syphilis diagnosis in correctional facilities.

**Conclusions:**

The course enabled the development of a massive training model for various health professionals at all care levels and regions of Brazil. This is particularly important in a country with a continental size and a large health workforce like Brazil. As a result, social and prison health impacts were observed.

## 1. Introduction

The health of people in prisons is an emerging and increasingly significant topic, despite the remaining negligence in many countries in terms of human rights, universal right to health, promotion of healthy states, and disease prevention in specific populations (1). The rising interest in prison health may be explained by escalating health problems in custodial settings, which has become a public health issue. The state of prison health has also deteriorated due to the growth of the prison population worldwide (1, 2). In most recent decades, such an increase, added to escalating health problems, has drawn global health attention (3).

Globally, the prison population has already surpassed 10 million people, with current data indicating a worldwide growth of 20% since 2000 (2). This scenario is striking as it is two percentage points higher than the estimated increase for the general population (18%) considering the same time period (1, 2). Hence, prison population growth may have been rising more than the estimated worldwide population growth. According to United Nations estimates, there are nearly 144 people in prison per 100,000 population globally (1). Therefore, this issue cannot be neglected, for it also impacts public health.

On a global scale, it is necessary to consider that the prison population growth rate can vary according to country or region, not consistently increasing. Oceania is a case in point. There, a 60% plus rise in the prison population was observed, while in the Americas, it totaled 40%. By contrast, a 21% drop was reported in Europe (1).

On a global perspective, in absolute numbers, the United States of America (USA) and China register the highest incarceration rates, with the prison population exceeding 2.2 million and 1.65 million persons, respectively. Meanwhile, Brazil and Russia have a prison population of over 600,000 people (2). The USA has one of the world's highest figures of incarcerated people per capita (698 persons per 100,000 population); Iceland holds the lowest rate worldwide, with 45 per 100,000 population (1), whereas Portugal's rate is 120 per 100,000 population (4). Of course, these data refer to social, political, religious, and ethnic determinants varying among regions and continents.

Given the world's prison population growth, health in custodial settings has become a major concern for health authorities, academic community, and other societal sectors, and it has been regarded as an urgent global health issue. This topic is included on the agenda of the United Nations Educational, Scientific, and Cultural Organization (UNESCO), the World Health Organization (WHO), and the Pan-American Health Organization (PAHO), which highlight the need for changes in prison system structures and health practices to comprehensively encompass the imprisoned population, the professionals operating in custodial settings, and the surrounding community (5–7). In this way, prison health has gained space and relevance in the strategic public health agendas.

Providing health care in the prison environment is, in effect, a way of guaranteeing the human rights of persons deprived of their liberty and, therefore, of the community into which they are integrated, as the latter is directly and indirectly affected by health problems inherent to this environment. That is why prison health implies to consider prisons and the territory in which they are placed, including imprisoned persons, their families, custodial staff members, and the surrounding community interacting with this system (8–10).

In this sense, strengthening universal health is paramount; and this can be achieved through a policy to reduce social inequalities that encompasses the goals set forth in the UN 2030 Agenda for Sustainable Development. This agenda sets forth 17 Sustainable Development Goals (SDGs) for people and the planet (11–13). In line with its plan of action, focusing on the 10 million people deprived of their liberty is vitally important to ensure human rights in societies that are just and grounded on sustainable development for all. Thus, one of the core principles of the 2030 Agenda is observed, which is that no one be left behind.

Projects that devote efforts to the prison community engage in an important social justice issue. Initiatives targeting well-being in health and health education for people in prisons and the prison community are considered fundamental by several international institutions and have been addressed in a series of studies and projects over recent years (14, 15).

One of these projects has been developed in Portugal by the Open University, within the scope of the governmental initiative Portugal INCoDe.2030, which has developed training activities in Health Literacy. Its main goal is to enhance the health literacy levels of imprisoned people, thus strengthening their abilities to care for themselves (4).

Brazil has one of the largest concentrations of people in prison globally, and presently, over 600,000 persons are deprived of their liberty (16). Prison health in this country is a public health emergency as disease prevalence has been increasingly worse, especially for sexually transmitted infections (STI) (17–22).

Several strategies and public policies aimed at the prison system have been established in Brazil. For instance, the Criminal Enforcement Law (CEL), No. 7,210, of July 11, 1984, is an important landmark. It addresses the rights of those deprived of their freedom in Brazilian correctional facilities and the necessary social reintegration.

The CEL shifts the prison system logic from a punitive system, *per se* and only, to a social reintegration model. Yet there is still much work to be done to effectively guarantee the rights of imprisoned people, especially in terms of access to health services. It should be noted that in Brazil, as in some other countries, the prison system can also be a place of social stigmatization, a fact that poses multiple challenges (23).

In order to comply with legal dictates, the Brazilian Ministry of Health (MoH) has laid forth public policies oriented toward the Prison System, thus including the topic in the national health agenda. Learning with an emphasis on prison health is one of the policy-inducing instruments that the Brazilian National Health System (SUS) uses in continuing education for healthcare professionals. Nevertheless, when it comes to a country with such a vast territory and regional diversities as Brazil, that constitutes a significant challenge.

Brazil, a continental-size country, has 26 states plus the Federal District and over 200,000 health care facilities in more than 5,700 municipalities. The country's health care workforce surpasses 3.5 million professionals working in a variety of jobs (24). In these circumstances, implementing a continuing education program with primarily in-person instruction, designed to foster rapid health system responsiveness, seems insufficient, mainly because of scalability matters and the urgent need for educational offerings.

To tackle scalability-related issues and meet SUS continuing education demands, the country has adopted massive health education strategies. This process has typically occurred by offering technology-mediated self-learning courses (25). Of note, the Virtual Learning Environment of the Brazilian Health System (AVASUS) has been successfully used in this context to provide prison health-oriented learning pathways (25–28).

While understanding the relevance of massive health education, especially as to tackling urgent prison health issues, this paper presents the results of a research study that assessed the reach of the online course “Health Care for People Deprived of Freedom” (ASPPL), available at: https://avasus.ufrn.br/local/avasplugin/cursos/curso.php?id=114. The ASPPL course was massively offered through AVASUS for all kinds of healthcare professionals across the Brazilian territory (18, 26). In addition to the results presented, we also provide a discussion and analysis grounded on publicly available epidemiological data from the Brazilian Prison System. Therefore, the primary goal of this study was to examine the aspects associated with massive education through technological mediation and its impacts on prison health.

## 2. Methods

### 2.1. Virtual learning environment of the Brazilian health system (AVASUS)

AVASUS is part of a technological ecosystem designed to provide continuing education for Brazilian healthcare workers, although the general population and people from other countries can enroll in educational offerings available. For instance, during the COVID-19 pandemic, AVASUS was highly demanded and reached 55 countries across all continents (29). Other notable AVASUS activities include training Community Health Agents in rural Tanzania, Africa (30). Effectively, AVASUS consists of “a virtual learning space for healthcare professionals, students, and general society to enhance SUS training, management, and care” (26, 31).

From 2015 (year of its launch) to 2022, AVASUS has contributed to the education of health workers throughout some major public health crises nationwide. Notable examples are the Zika Virus (agent that causes microcephaly) crisis in Brazil in 2015, the syphilis epidemic, ongoing since 2018, and, more recently, the COVID-19 pandemic (2020–2022) (31). COVID-19-related courses alone accumulate over 300,000 enrollments (26).

This virtual learning environment accumulates over 840,000 participants who enrolled more than 2.16 million times in the 318-plus courses. This means that the enrollment rate is close to 2.5 courses per participant (31). Moreover, more than 1.3 million certificates of completion were issued to all participants who completed AVASUS courses. The figures are publicly available at: https://avasus.ufrn.br/local/avasplugin/dashboard/transparencia.php.

Recognizing the necessity of qualifying health professionals to address the problem of health in the prison system–especially as imprisoned persons are among the key populations for syphilis infection in Brazil—, the ASPPL course was developed and has been offered in the learning pathway “Syphilis and Other STI” (26). The goal was to enhance the response to syphilis infection in the Brazilian prison system through a massive health education process.

As the course uses a dialogical education process through technology mediation that AVASUS articulates, it also provides participants with opportunities to develop a variety of competencies necessary to ensure comprehensive health care to the imprisoned person—with interference in expanded social understandings of life and its helplessness (26).

### 2.2. Study design and participants

This is a cross-sectional study of the course “Health Care for People Deprived of Freedom,” offered on AVASUS since June 2018 (https://avasus.ufrn.br/local/avasplugin/cursos/curso.php?id=114). Recently, Valentim et al. (18) published a Data Report on the course. Such a significant scientific contribution allowed other researchers to undertake more detailed studies. However, the work cited provides a brief description of the results without delving into their impact on prison health, aspects fundamental in measuring the intervention and promotion of health policies in the prison system, according to the principles of Permanent Health Education (EPS).

Allen et al. (32) argue that impact evaluation from EPS actions should far transcend course outcomes measurement regarding the quality of education. For (33), understanding and evaluating the context is necessary and should be based on the question, “How/why did the program work, and what else happened?”

Therefore, in an addendum to the Data Report database by Valentim et al. (18), we have expanded the sample from 4,861 to 8,118-course participants (duly anonymized population) who enrolled until January 14, 2022. In addition, for context analysis to support the discussion on the impacts of the course on prison health, we included epidemiological data relative to syphilis in Brazil and the country's prison system. All data were retrieved from public databases.

### 2.3. Data acquisition

Data were collected from six public sources. These are as follows: (1) AVASUS; (2) Brazilian National Registry of Health Care Facilities (CNES); (3) Brazilian Occupational Classification (CBO); (4) National Prison Department (DEPEN); (5) Brazilian Institute of Geography and Statistics (IBGE); and the (6) Brazilian Ministry of Health (MoH), through the Outpatient Information System of the Brazilian National Health System (SIA/SUS). Of note, all data from the various sources were duly anonymized and made available in a repository of public domain (open data, available at: https://zenodo.org/record/6499752#.YmoLffPML0r). As this study did not involve experiments on human beings, approval by a research ethics committee was not required.

We collected data from 8,118 enrollees in the ASPPL course from AVASUS records (34). A total of 106 characteristics were extracted from these data, which included the main attributes we analyzed, namely gender, region, CNES, CBO, course completion percentage, and course evaluation. It should be noted that the ASPPL course receives applications on a rolling basis. Thus, the data covers the period between the course launch date (7 June 2018) and the final data collection date (14 January 2022).

These data are strategically important in identifying the profiles of course participants, i.e., health profession, type of healthcare facility, and the Brazilian region of workplace. Additionally, it is possible to verify at which levels of the SUS network health workers are operating (primary, secondary, or tertiary care).

In contextual analysis, five different sources were used to build a cross-sectional dataset, which did not exclusively contain the AVASUS database. Sources included CNES (35), CBO (36), DEPEN (16), SIA/SUS (37), and IBGE (38). CNES and CBO data were used to identify the profession and professional affiliation of course participants within Brazilian health facilities. After processing such information, we integrated CNES and CBO data with participants' data retrieved from AVASUS. Hence, data from heterogeneous sources were incorporated into a single database.

To link data between AVASUS and CNES data, a shared ID (Proof of Registration Status) was used in both databases. In the same way, data linking between CNES and CBO data was achieved through the occupation code found in both databases.

To underpin research analysis, data on each Brazilian region's population and the prison system were compiled from the Public Data Portal of IBGE and DEPEN, respectively. Furthermore, prison system epidemiological data were collected from DEPEN, including data on the number of serological tests performed to detect syphilis in all five Brazilian regions. The reference period of these data covers 2017 to 2020 (see **Tables 2**, **3**).

### 2.4. Data processing

After data acquisition, a pipeline (or workflow) was defined and executed to integrate, transform, and organize the data for study analysis and publicize the dataset to the scientific community. The data processing pipeline was done using the Python programming language (3.8.9), including its best-known data manipulation and visualization libraries (pandas, NumPy, re-regular expression, Matplotlib, and seaborn). It consisted of a three-step process: (i) data integration and standardization, (ii) feature extraction, and (iii) feature selection.

In step (i), the descriptive classification of the occupations of course participants working in SUS was integrated into the main dataset. This procedure was accomplished through the CBO codes–initially collected through AVASUS and then verified and updated in the CNES database. The descriptive classification was imported after querying the official CBO database (36).

Also, in step (i), health facility data were integrated, namely data on the levels of the SUS healthcare network, where course attendants are employed. In this very step, some database attributes underwent a normalization process. Effectively, this standardization was necessary for the analyses to be carried out because each data source uses different nomenclatures. For instance, participants' gender was standardized to “Female,” “Male,” and “Not Informed.” Moreover, we have also standardized the attribute relative to the participants' States (UF, Federative Units of Brazil).

For the CBO attribute, participants who had no official professional affiliation were labeled as “individual without official affiliation.” A special treatment, based on the regular expression method, was performed in the descriptive attribute of participants' professions to minimize the dispersion among derived ones. A good example is the different occupation descriptions derived from the medical field of knowledge (i.e., medical specialists), which were treated and clustered into the single group “Physician.”

Step (ii), feature extraction, exclusively focused on creating new attributes related to participants' health facilities and regions. Firstly, based on the students' CNES, attributes associated with levels of care in Brazil were created. These are: “primary health care” (PHC), “medium complexity” (secondary health care), and “high complexity” (tertiary health care).

These attributes contain Boolean values and indicate participants' professional practice affiliation in Brazilian health services. The attribute “region” was created so that participants could be grouped according to their region in Brazil: North, Northeast, Midwest, Southeast, or South. In this instance, the attribute “region” was based on IBGE's political-administrative and regional division of Brazil (39). Therefore, for this grouping, it was used the attribute referring to the attendee's State included in the AVASUS dataset.

The feature selection step (iii) defined the main attributes used in this study's analysis. In addition, this step was fundamental in ensuring that the dataset was consistent, coherent, anonymized, and suitable for publication in a public repository. A detailed description of the “asppl_dataset_v2.csv” dataset is available for public consultation at https://doi.org/10.5281/zenodo.6499752.

### 2.5. Data analysis

The data analysis was based on statistical tools such as mean, median, and standard deviation. Absolute numbers of grouped data with their respective equivalent percentages were also examined. In addition, Equation (1) (see notations) was used to normalize data for comparison purposes between each region and Brazilian populations. Therefore, the coefficient (*rate*) accounts for the proportion of each population being studied (normalized values).

**Figure 1:**
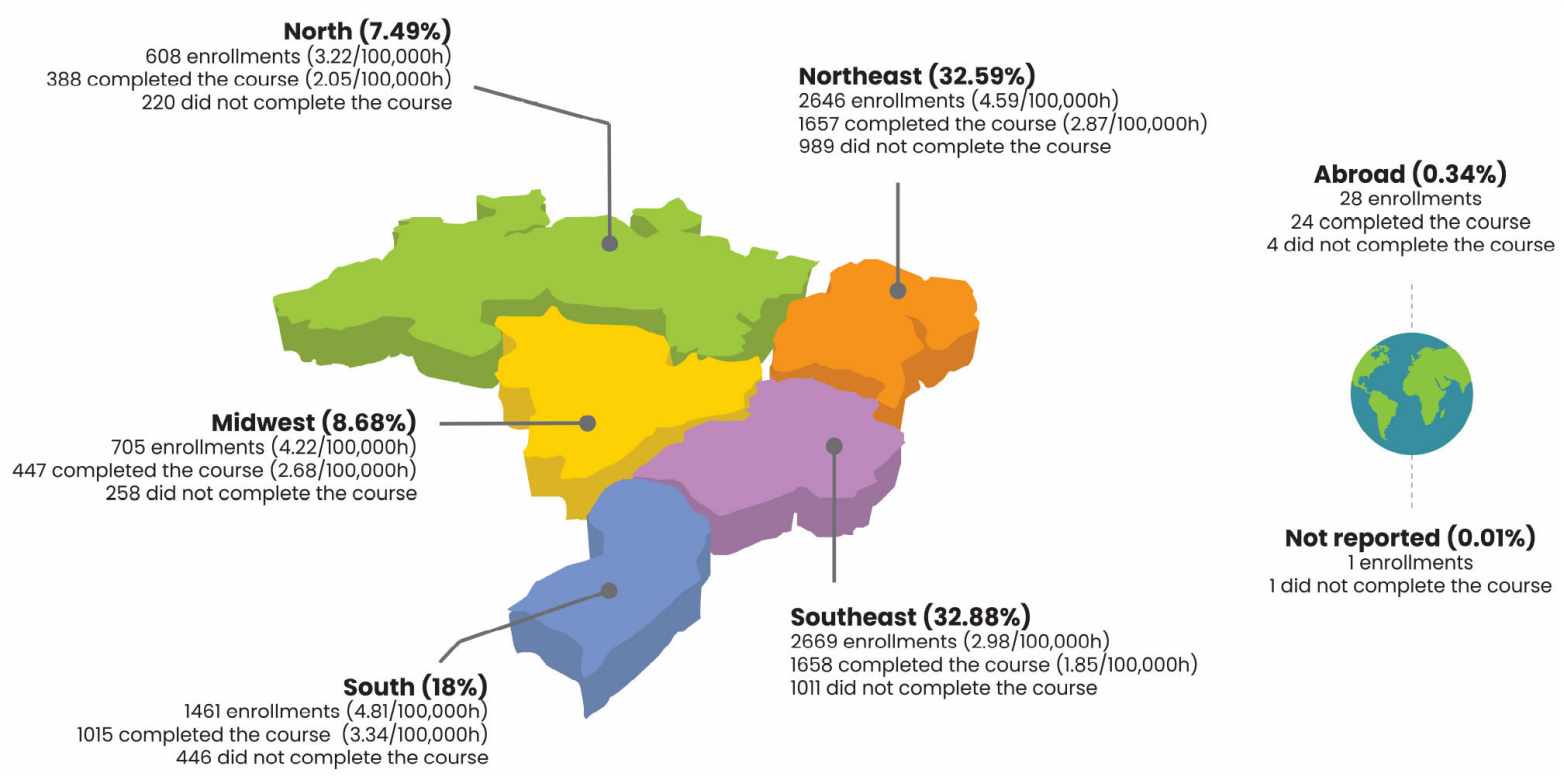
Analysis of course participants and enrollments by Brazilian region.

Equation (1) was primarily used in generating **Table 3**, aside from calculating the ASPPL course enrollment and completion rate (Figure 1). Thus, population estimates of Brazilian regions, prison population, number of students enrolled in the ASPPL course, the total number of syphilis infections among the imprisoned people, and serological syphilis tests performed in prisons were considered. The following notations were defined for the variables included in Equation (1):


(1) $$\begin{array}{r} rate=\left(\frac{x_{target}}{x_{pop}}\right)n_{factor} \end{array}$$

were:

– *rate*: variable used to store the coefficient relative to indicators proportional to each region or the country
– *x*_*target*_: variable used to determine the value associated with indicators (number of students, syphilis tests, syphilis in prison)
– *x*_*pop*_: variable used to determine the value relative to the population of each region
– *n*_*factor*_: variable used to determine the proportionality factor.

## 3. Results

From 7 June 2018 to 14 January 2022, the ASPPL course had 8,118 enrollments from participants from all five Brazilian regions. Figure 1 summarizes characteristics that reveal the course amplitude, such as the spatial distribution of course participants across Brazil, enrollments, and completion rates per 100,000 population (see Equation 1), and the absolute number of enrollees who have completed the course per region. Note that, in addition to people from the five Brazilian regions, 28 participants abroad have enrolled in the course being alluded to, which corresponds to 0.34% of total enrollments.

According to Figure 1, it is apparent that the Southeast and Northeast regions hold the highest number of enrollment (in absolute numbers), 2,669 (32.88%) and 2,646 (32.59%), respectively. Yet, in proportional terms, the South stands out clearly as the state with the highest count of students enrolled and students who have completed courses per 100,000 population: 4.81 and 3.34%, respectively. In line with the proportional analysis, the Northeast and Midwest have values comparable to the South region, when considering enrollment rates. The South region has the highest course completion rate, followed by the Northeast (see Figure 1).

Overall, the ASPPL course qualified 5,190 (63.93%) participants. This count includes all five regions, enrollees abroad, and those who did not report their location. Of those, 4,752 (91.56%) filled up a course evaluation, assigning a score ranging from 0 to 5 derived from their level of satisfaction. Thus, the course obtained a 4.94 mean evaluation score and reached the highest median score, 5.0. The standard deviation (SD) was 0.35. Both scores strongly indicate that students positively evaluated the course.

**Figure 2:**
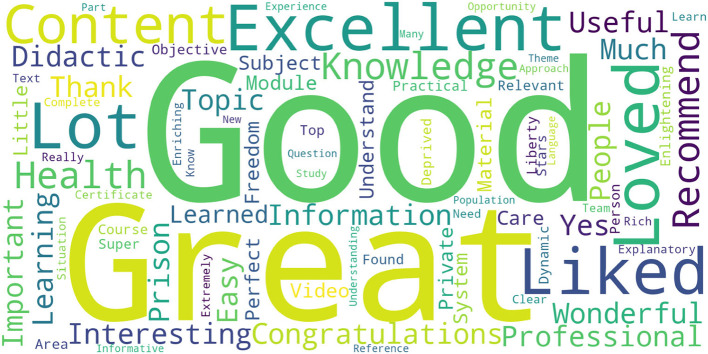
Word cloud of participants' comments.

Figure 2 shows a word cloud that corroborates this information. The most frequent words participants typed during evaluation—the moment at which they complete the course—define their feelings toward the quality of the course (most frequent words are good, great, excellent, and liked). We prepared this word cloud from texts that the attendees wrote spontaneously (they were not pushed to write them) after evaluation of course quality.

### 3.1. Characterization of course participants

**Figure 3:**
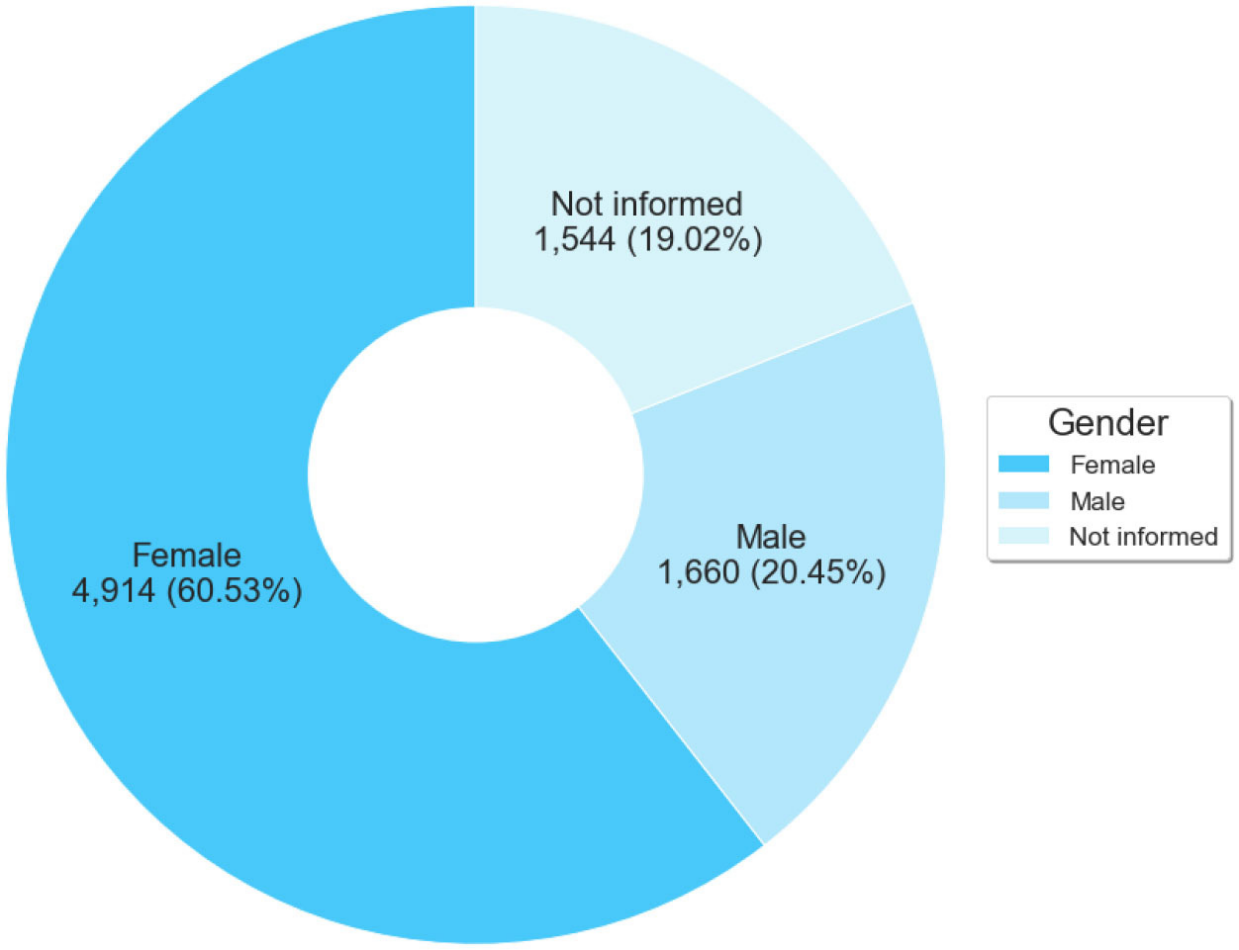
Number of participants by Gender.

This characterization provides the profile of participants and their place of work. First, we present Figure 3 to highlight the prevalence of the female gender among ASPPL enrollees, which recorded 4,914 participants (60.53%). The remaining participants were males and other people who did not inform their gender: 1,660 (20.45%) and 1,544 (19.02%), respectively.

**Figure 4:**
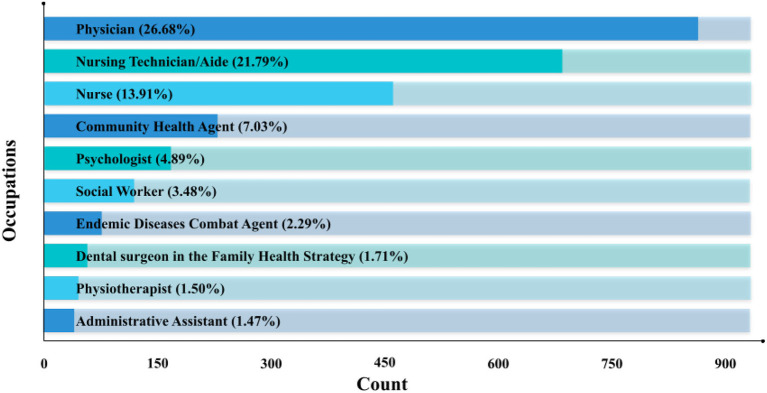
Distribution of occupations among course participants.

As for the occupation or profession of the participants, 3,272 (40.31%) declared their CBO code or had this number identified through CNES. Figure 4 breaks down, both descriptively and quantitatively, the ten most frequent occupations among participants. From this group of 3,272 professionals, the four most frequent occupations stand out, with 873 (26.68%) attendees working as physicians, 713 (21.79%) as nursing technicians or nursing aides, 455 (13.91%) as nurses, and 230 (7.03%) as community health agents.

Of the 3,272 professionals, 2,009 (61.40%) evaluated the ASPPL course. Table 1 lists the ten occupations with the highest number of evaluators and the evaluation metrics values. Considering the overall perspective of this subgroup of professionals, the evaluations mean was 4.92 (median = 5.0, SD = 0.39).

**Table 1:** Evaluation by course participants according to occupation.

**Occupations**	**Number of evaluations**	**Evaluation metrics**		
		**Mean**	**Median**	**SD**
Physician	623	4.89	5	0.42
Nursing technician/Aide	473	4.92	5	0.41
Nurse	257	4.89	5	0.48
Community health agent	141	4.96	5	0.22
Psychologist	79	4.96	5	0.19
Social worker	55	4.98	5	0.13
Endemic diseases combat agent	45	4.97	5	0.14
Dental surgeon in the Family Health Strategy	40	4.92	5	0.47
Oral health assistant in the Family Health Strategy	30	4.96	5	0.18
Physiotherapist	26	4.96	5	0.19

Considering all 8,118 participants, it is worth noting that 4,846 (59.69%) stated not to have an official affiliation, i.e., they reported having not currently or previously worked in any occupation at the time of course attendance. It is possible that these students without official CNES affiliation are health students or people from the general population interested in the topic.

**Figure 5:**
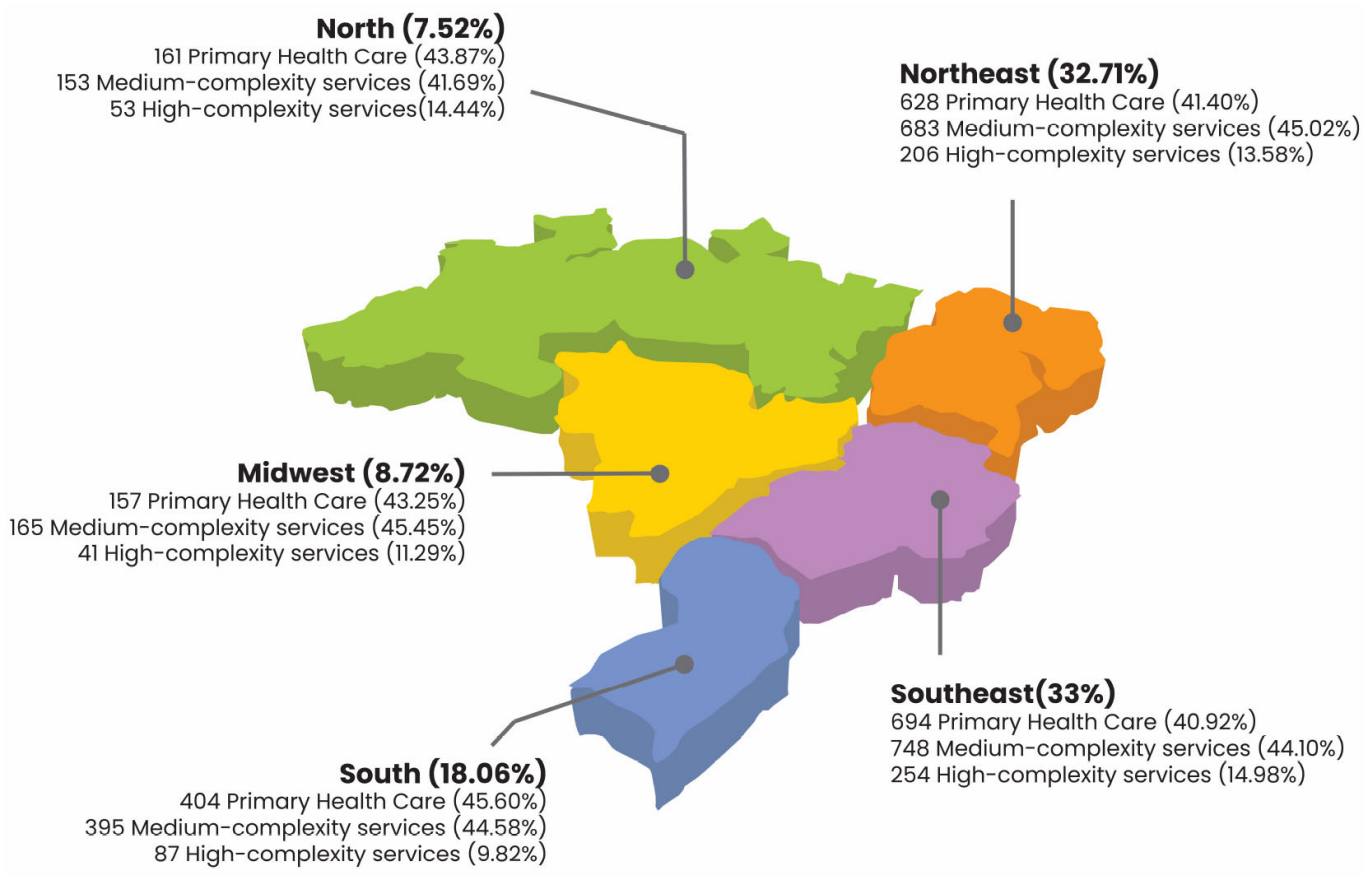
Trajectory of healthcare professional attendees by level of health care and region.

Brazil follows the World Health Organization (WHO) classification for levels of health care, which defines three different levels according to the complexity of care: primary, secondary (medium complexity), and tertiary health care (high complexity). Figure 5 provides an overview of the geographical region of course participants who work in SUS and in which health care levels they operate.

According to Figure 5, participants who enrolled in the ASPPL course work in different health care settings, regardless of Brazilian region. It is noteworthy that, in absolute numbers, participants from the SUS workforce from all five regions operate mainly in facilities that provide health services in primary and/or secondary care. This could be explained by the fact that these two levels of health care networks are more directly related to health in the prison system.

In Brazil, one of PHC attributions is to operate in the community; therefore, prison health is also included in such attributions. Meanwhile, the secondary care network (SUS medium complexity services) offers outpatient specialty or reference services, which are also directly connected to prison health.

## 4. Discussion and analysis

The present section considers the context of massive health education for the Brazilian prison system, as well as aspects related to this system and epidemiological issues. Therefore, we not only sought to answer questions about the results relative to the ASPPL course but also to examine how the training process potentially made an impact on prison health.

Imprisoned persons are vulnerable to communicable diseases such as syphilis, HIV, tuberculosis, and hepatitis (19, 40, 41). Hence, they are also a key population for public health policies addressing conditions alike. Caring for prison health means caring for the community or the general population's health because generally, the prison population is not referred to a hospital or outpatient health service, listed as having or suffering from certain illnesses, nor have their data aggregated in epidemiological indicators. Therefore, becoming ill or having health compromises in custodial settings is a direct result of a combination of factors—e.g., imprisonment conditions, lack of access to information or care, and lack of programs and policies aimed at the prison population—, not to mention the fact that it represents risks and diseases profiles across the community. The opposite is also true.

Prisons entail a severe public health problem and pose challenges to global health. Watson et al. (3) state that “Health promotion and the health of the community outside prisons are desirable aims of prison health care. The delivery of effective health care to prisoners is dependent upon a partnership between health and prison services.” In Brazil, that is no different. The increase in prison population puts the country in a negative ranking, as it figures in top positions among countries that imprison the most people globally. This reflects the country's social inequalities, which perpetuate inequities and, consequently, imprisonment and a higher burden of disease in prisons.

Table 2 shows that this phenomenon occurred in all country regions except the Southeast region, where a drop was reported in 2020. In this instance, it could be explained by the COVID-19 pandemic, when the Supreme Court of Brazil authorized the temporary release of imprisoned persons in high-risk groups, releasing ~30,000 people (42). Finally, the Southeast Region holds the largest prison population, with ~46% of imprisoned people (see Table 2).

**Table 2:** Population of Brazil and the prison system per region and year.

**Region**	**Population**				Population in prison (PoP)^*^			
	**2017**	**2018**	**2019**	**2020**	**2017**	**2018**	**2019**	**2020**
North	17,929,800	18,182,253	18,430,980	18,672,591	52,167	57,414	63,346.5	64,980
Northeast	56,442,149	56,760,780	57,071,654	57,374,243	119,835.5	126,835	137,397.5	140,159.5
Midwest	15,870,886	16,085,885	16,297,074	16,504,303	66,093.5	68,497	71,909	85,226
Southeast	87,035,037	87,711,946	88,371,433	89,012,240	373,028.5	380,846	385,399	359,190
South	29,526,869	29,754,036	29,975,984	30,192,315	94,473	84,852	92,091	131,130.5
Brazil	206,804,741	208,494,900	210,147,125	211,755,692	705,597.5	718,444	750,143	780,686

* Notation: N = {2017, …, 2020}. Calculation: $PoP_{i}^{r}=\frac{PoP{\left(jan-jun\right)}_{i}^{r}+PoP{\left(jul-dez\right)}_{i}^{r}}{2},\;\forall i\;\in N$ and ∀r ∈Region.

In 2016, Brazil declared a syphilis epidemic when a nearly 5,000% rise in syphilis infections was observed. In 2017, the MoH introduced a national policy to fight the disease in the country (43). Such a policy includes key populations and prioritizes the prison population.

Then, in 2018, the ministry launched the “Syphilis No!” Project (SNP) as part of an interfederative pact between the MoH, the State Health Secretariats (there are 26 across Brazil, plus the Federal District), and the Municipal Health Secretariats (there are 5,570 distributed over the 26 Brazilian states, plus the Federal District) (44). As a result, for the first time in over 30 years, the topic of syphilis was included in the national public health agenda, a fact that mobilized several initiatives through a national pact (interfederative actions between the Union, States, and Municipalities). Syphilis in Brazil stopped being a neglected disease after at least three decades (45).

The SNP planned universal and local actions and was one of the tools to spur the national public policy to fight syphilis in the country. Local actions were carried out through direct public health interventions in 100 municipalities considered priorities—chosen by the MoH for having the highest syphilis incidences in Brazil). Universal actions included the distribution of penicillin, syphilis testing, public health communication campaigns, and health education (46, 47).

In the health education dimension, an educational pathway with more than 50 courses on syphilis and other STI was designed, produced, and offered through AVASUS. Of note, the course ASPPL is the only course in this educational pathway that aims to qualify health professionals on this topic (26).

This contextualization of syphilis in Brazil helps to explain the course's activities and educational proposal. The goal of the ASPPL course was to promote massive and nationwide training by reaching the largest number of people, particularly health professionals operating in SUS health facilities. This process occurred spontaneously because none of the professionals were forced to enroll in and take the course. This was only possible because AVASUS—which has national coverage and operates through technological mediation—was used and constituted a determining factor in achieving the necessary scalability in the face of the public health urgency caused by syphilis in Brazil.

Over the time period this study covers, 8,118 participants enrolled in the ASPPL course, of which 5,190 (64%) have completed it. This massive training strategy can be considered successful since the course achieved a high completion rate in all Brazilian regions, with ~193 participants per month and 2,139 per year. Moreover, the fact that 36% of students had not yet completed the course is not considered a problem as the course is self-regulated (the participant directs their course of study). In addition, some enrollees are only interested in taking specific course units, an aspect that was not evaluated in this study as AVASUS does not yet provide micro-certification.

Table 3 breaks down epidemiological data and shows the rate of participants per 100,000 population and the rate of syphilis testing and syphilis infections per 1,000 population (in relation to the prison population), wherein data normalization is essential for comparisons among the regions and the country itself. Table 3 also depicts relevant data showing a growth trend in all regions when the rate of students per year is considered. Even though the Southeast and North regions hold a lower rate of participants compared with the national rate, it has a growth trend. This factor is positive because it evidences that the interest in the topic has not lessened, even during the pandemic, as some experts had predicted that the great deal of global effort to combat COVID-19 could strain health care networks aimed at STI control (48, 49). One possible explanation for enrollment rate results is the mass delivery model used, enabled by technological mediation.

**Table 3:** Summary of the ASPPL course indicators, syphilis tests performed, and syphilis in the prison system.

**Region**	**Students (** ***rate*** **)^a^, ^b^**			**Syphilis tests (** ***rate*** **)** ^*****c*****^				**Syphilis in prison (** ***rate*** **)^c^**			
	**2018**	**2019**	**2020**	**2017**	**2018**	**2019**	**2020**	**2017**	**2018**	**2019**	**2020**
Northeast	537 (0.95)	849 (1.49)	1,204 (2.1)	3,652,437 (64.71)	5,116,669 (90.14)	4,758,341 (83.37)	3,987,258 (69.5)	1,062.5 (8.87)	1,353.5 (10.67)	1,541 (11.22)	1,052.5 (7.51)
Southeast	463 (0.53)	737 (0.83)	1,141 (1.28)	4,023,328 (46.23)	6,000,901 (68.42)	5,109,036 (57.81)	5,140,036 (57.75)	2,198 (5.89)	2,334 (6.13)	2,748 (7.13)	2,007.5 (5.59)
South	278 (0.93)	404 (1.35)	674 (2.23)	1,521,658 (51.53)	2,449,347 (82.32)	2,313,173 (77.17)	1,331,642 (44.11)	1,037.5 (10.98)	681.5 (8.03)	710.5 (7.72)	811 (6.18)
Midwest	135 (0.84)	192 (1.18)	284 (1.72)	1,010,069 (63.64)	1,359,956 (84.54)	1,520,205 (93.28)	762,879 (46.22)	438.5 (6.63)	762 (11.12)	876 (12.18)	448 (5.26)
North	131 (0.72)	188 (1.02)	252 (1.35)	1,590,936 (88.73)	2,322,215 (127.72)	1,872,062 (101.57)	1,171,197 (62.72)	392 (7.51)	435.5 (7.59)	559 (8.82)	355 (5.46)
Brazil	1,544 (0.74)	2,370 (1.13)	3,555 (1.68)	11,798,428 (57.05)	17,249,088 (82.73)	15,572,817 (74.1)	12,393,012 (58.52)	5,128.5 (7.27)	5,566.5 (7.75)	6,434.5 (8.58)	4,674 (5.99)

a The cumulative sum of students enrolled in the current year plus previous years.

b From Equation (1): n_factor_ = 100, 000.

c From Equation (1): n_factor_ = 1, 000.

It is essential to consider that in a continental-size country like Brazil, which faces great social inequalities and is still immersed in the COVID-19 pandemic, it would be highly complex to use face-to-face models to train health professionals on prison health at scale. Therefore, when this context is analyzed, the relevance of massive education with technological mediation is apparent. In effect, this model tends to be more appropriate during a health crisis. In the case under study, Brazil was undergoing two simultaneous crises, i.e., syphilis epidemic and COVID-19 pandemic. This model, using a massive and scalable education model structured through technological mediation, managed to foster more resilience in the context of prison health education. Otherwise, it might not have achieved such a broad reach.

Faced with the COVID-19 pandemic, the continued interest in the topic of syphilis in Brazil is explained by the interventions developed by the SPN nationwide (47), added to other actions articulated through the MoH, states, and municipalities. Therefore, it is reasonable to assert that in Brazil, during the COVID-19 pandemic, efforts to promote health policies in response to the syphilis epidemic have not ceased. For example, the communication actions of the SNP contributed to the topic being steadily prominent in the public health agenda of states and municipalities (47). In addition, a campaign to promote the courses of the educational pathway “Syphilis and other STI” (this includes the ASPPL) was carried out through a set of interfederative actions, especially in the 100 priority municipalities (26, 46).

The study by Rocha et al. (45) reinforces such an idea and presents quanti- and qualitative data to show that intervention actions in priority municipalities have set the syphilis issue in the public health agenda, and these actions occurred even under an epidemic. This aspect also helps explain the trend of increased enrollment in the ASPPL course during the COVID-19 pandemic. Simultaneously, it reinforces that promoting public health policies is part of a relevant strategy that imbues the health system with better resilience and responsiveness (29). In this instance, such as that of technology-mediated education in Brazil's prison system.

In Table 3, the column “Syphilis Test” indicates a significant increase in syphilis testing in Brazil in 2018, when the SNP launched. Table 3 also provides data for 2019 and 2020, where a decrease in syphilis testing was observed. However, it remained higher compared to 2017. Note that a similar trend was observed at the national level. The year 2020 should be specially highlighted, for that is when testing could have decreased sharply due to the pandemic. However, that did not occur compared to 2017, prior to COVID-19.

According to Rocha et al. (45), Pinto et al. (46, 47), and Valentim et al. (26), this phenomenon is explained by the SNP interventions, both in the dimensions of health communication and health education. The authors evidenced associations between public health interventions, increased testing uptake, and a decline in syphilis infections in Brazil, particularly congenital syphilis cases. These data are essential for analyzing the numbers shown in the “Syphilis in Prison” column (see Table 3).

According to the figures in the same column (Table 3), the number of syphilis cases in the Brazilian Prison System has noticeably increased between 2018 and 2019. In both years, the count of reported cases was higher than in 2017. This stems from the notable increase in testing in Brazil; ~33 million syphilis tests were performed over this period. Increased testing improves diagnosis, a factor that induces treatment (testing and timely treatment, and in the case of syphilis, cure). Syphilis testing and treatment are vital to breaking the chain of infection.

The data relative to the rate of courses in the “participants” column and those in the “Syphilis in Prison” column reveal that, in 2018, the regions with the highest course completion rate held the highest rates of syphilis in the prison system. Namely, the Northeast, South, and Midwest regions. The increase in reported cases is not regarded as a problem, as it may indicate better testing in the prison system in such areas, thereby increasing case reports.

The fact that there are no reported cases of syphilis in prisons does not mean that they do not exist. Southeast Brazil is another region that drew attention as, in 2018 and 2019, this region recorded the lowest rate of participants. Nonetheless, this region also recorded the lowest rate of attendees who completing the course. Although it is not yet possible to establish a causality relation, these data should be considered in any analysis since health education is paramount in inducing responsible and competent responses in health systems hence this would be no different for the prison system (29, 50, 51).

**Figure 6:**
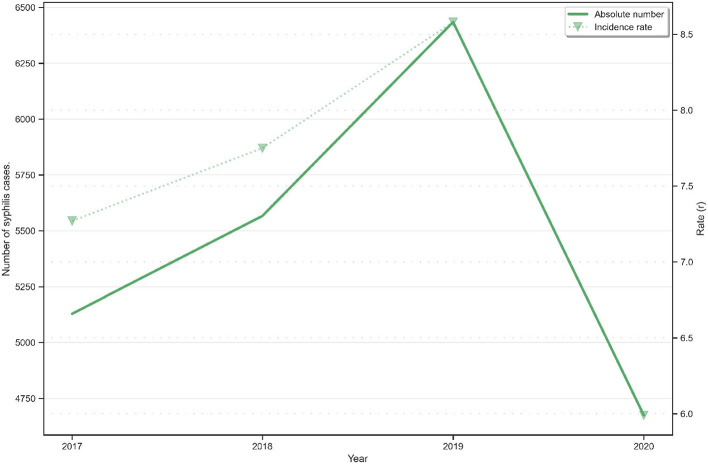
Syphilis infections in the Brazilian Prison System and rate per 1,000 population (in relation to prison population).

Regarding the data on syphilis cases within the prison system between 2017 and 2019, an inflection point in 2018 is notable (see Figure 6). It shows a significant rise in case numbers of nearly 15% between 2018 and 2019. There has also been a 53% increase in the number of participants completing the ASPPL course during the same period. Taking into consideration the increase in cases between 2017 (the year when the ASPPL course had not been launched on AVASUS) and 2018, it can be seen that there was pronounced nominal growth (8.5%) in case reporting compared to the 2018–2019 period.

Two key aspects should be considered in this analysis. First, 2018 was when Brazil recorded the highest number of tests in all regions, so it would have been the year in which the highest number of cases reported in the prison system would appear in the records compared to 2017. However, this rise did not occur. Second, fewer syphilis tests were performed in 2019 compared to 2018; thus, it should have been reported fewer cases of syphilis than in 2018. However, this did not occur. In this same period, though, AVASUS recorded a 53% increase in participants completing the ASPPL course.

The increased number of completion and the higher rate of syphilis cases reported in prison indicate two shreds of evidence: a spontaneous increase in the educational demand on the topic since the participants were not forced to enroll in the course; and, simultaneously, a change in work processes, given that the increase in case notification is determined by the increase in testing in the prison system. According to the Syphilis Epidemiological Bulletin, released in 2021 by the MoH, Brazil recorded a case drop for all types of syphilis (acquired, maternal, and congenital) in 2019. However, that did not occur in the Prison System because, in 2019, Brazil recorded the highest surge in cases, which shows an increase in diagnosis (testing) in this environment, concomitant to the rise in participants completing the ASPPL course.

The analyses presented point to the effectiveness of massive health education; in this instance, the object of study was the course “Health Care for People Deprived of Freedom,” offered through AVASUS for health professionals across all regions of Brazil. The data analysis also shows that the ASPPL course, apart from qualifying more than 8,000 health professionals, proves to be a strategic and relevant tool for public health policy interventions for the prison population, as it has presented results that can induce resilience and improve the response to the problems posed by syphilis in prisons.

Regarding 2020, one must consider the effects of the COVID-19 pandemic concerning case reports. Nevertheless, the number of syphilis tests performed in Brazil in 2020 was higher than in 2017. Yet, the number of syphilis cases in the prison system was lower in 2020. In this context, it cannot be said that infections were underreported when, in fact, there was a higher number of syphilis tests in the country compared to 2017. One aspect that can be considered to justify this reduction in syphilis cases in prisons in 2020 is the expressive increase in diagnosis in 2019 (notifications), which may have contributed to reducing the chain of transmission. In this case, it is essential to highlight that, in 2020, AVASUS recorded a 48% increase in participants completing the ASPPL course compared to 2019, i.e., it maintained the trend of spontaneous growth. This aspect reflects an interest in the course.

## 5. Considerations

The main innovation of this work emerges from the development of an impact analysis of massive health education through technological mediation on the prison system. The number of students reached nationwide, coupled with the increase in syphilis testing within the Brazilian Prison System, as presented in the results, demonstrate that improvements in the work processes have been made. Therefore, these results underline the importance of qualifying the health workforce in the dimension of prison health.

Cross-referencing educational and epidemiological data in the context of prison health is not trivial; however, it has contributed to demonstrating that massive health education fosters health resilience in the prison system. In line with the 2030 Agenda, our approach sheds light on an emerging topic of global public health interest and recognizes it as a priority, thereby enabling the analysis of social impacts beyond health.

The 2030 Agenda for Sustainable Development, adopted by all UN Member States in 2015, provides a shared blueprint for peace and prosperity for people and the planet, now and into the future. At its heart are the 17 Sustainable Development Goals (SDGs), which are an urgent call for action by all countries—developed and developing—in a global partnership. They recognize that ending poverty and other deprivations must go hand-in-hand with strategies that improve health and education, reduce inequality, and spur economic growth while tackling climate change and preserving our oceans and forests (12).

The course “Health Care of Persons Deprived of Freedom” encompasses the general characterization of the prison population, core public policies targeting this population, and relevant discussions for PHC professionals. PHC plays a critical role in shaping and promoting public health policies. This facilitates the management of appropriate health care, health promotion, and disease prevention among imprisoned people. Therefore, the ASPPL course acknowledges the breadth of the topic under discussion and brings attention to the healthcare needs of the prison population, while encouraging health professionals to engage with the subject.

Ismail et al. (11) emphasize that 9 out of the 17 SDGs are directly implied in the prison system, and eight, indirectly implied. Based on the SDGs directly implicated, we could observe that the course produces transversal impacts besides those analyzed in the Discussion section of this article. Such impacts are in synergy with at least five goals directly implicated in policies for the prison system:

**SDG 3**—Ensure healthy lives and promote well-being for all at all ages. This goal aggregates the 2010 “Bangkok Rules” and the 2015 “Nelson Mandela Rules” (11, 52, 53), which are existing policies to encourage changes in the field of Prison Health (54). Notably, prenatal and postnatal care and sexually transmitted diseases (STD) are highlighted. Unit III of the AVASUS ASPPL course, namely “Comprehensive Health Care for Women Deprived of their Liberty,” focuses on the main problems affecting women, the mother-child dyad in maternity situations, and the role of the Family Health Team (ESF) in welcoming and caring for imprisoned women. The theme of STDs, or STI, is a transversal throughout the course.
**SDG 4**—Ensure inclusive and equitable quality education and promote lifelong learning opportunities for all. This is a crucial goal or catalyst for the other goals throughout the 2030 Agenda, as Education has always been a foundational basis for societal change. This goal has the integration of policies (11): the 1985 “Beijing Rules”; 1990 “Havana Rules”; the 2010 “Bangkok Rules”; and the 2015 “Nelson Mandela Rules,” in unison to encourage and adapt educational and vocational guidance actions to provide opportunities for lifelong learning (52, 53, 55, 56). As a goal of this objective, we have “the quality of education personnel in prisons,” envisaging formative strategies for people operating in prisons. In this vein, the ASPPL course falls perfectly into a massive training and learning strategy for prison system personnel.
**SDG 10**—Reducing inequalities and ensuring no one is left behind are integral to achieving the Sustainable Development Goals. Empower and promote the social, economic, and political inclusion of all people, regardless of age, gender, disability, race, ethnicity, origin, religion, economic, or other status. This goal calls for “a life of equality for all people.” This directly includes persons deprived of their liberty, with the underlying thought that no one should be left behind. This goal also incorporates already existing prison system policies, which are: the 1985 “Beijing Rules” and the 1990 “Havana Rules” (55, 56). One of the objectives of the ASPPL course is to provide massive education for health professionals working in the prison system to promote changes in working behavior and professional practices, collectively and individually. To contribute to these changes in health workers' practices represents creating concrete possibilities for improvement in prison health. In addition, it means to ensure the right to quality health care for persons deprived of liberty.
**SDG 11**—Make cities and human settlements inclusive, safe, secure, resilient, and sustainable. Yet another Goal that highlights the policies of the Nelson Mandela Rules (53) for ensuring, even amid overcrowded prison systems, prisons that are resilient, safe, and sustainable. By reflecting upon the ASPPL course, the training for healthcare professionals working in prisons is observed again. Ensuring health and well-being in the prison system is to work toward a safer, more resilient, responsive, and sustainable system from a public health perspective. Therefore, it encompasses all actors involved: the prison population, prison officers, health and social services personnel, other workers involved, and the community (family members, social networks, and territory of social and community relationships).
**SDG 16**—Promote just, peaceful and inclusive societies, provide access to justice for all, and build effective, accountable, and inclusive institutions at all levels. This SDG, akin to Goal 4, brings together a multitude of existing policies for the prison system (11): the 1985 “Beijing Rules;” the 1990 “Havana Rules;” the 1990 “Beijing Rules;” the 2010 “Bangkok Rules,” and the 2015 “Nelson Mandela Rules” (52, 53, 55–57). The main concern is to guarantee the rights of people deprived of liberty through ethics and social justice, for more peace and less violence, and to address the health coefficients relative to violent behavior. In the ASPPL course, the unit on “Mental Health of People Deprived of Liberty” discusses the main demands regarding this issue in prisons and the role of the Family Health Strategy (ESF) in welcoming and caring for such matters.

Prison health is a topic with multifactorial problems. That is why recognizing the necessity of an integrated approach to public policy is key to discussions in this field. “No one left behind” (12); this is the main motto of Agenda 2030. With this sentiment and realization that the vast and increasing prison population results from global and health problems, it is necessary to look not only behind bars, but it is also essential to look beyond the concrete. Caring for prison health means caring for health in the community. Therefore, the importance of the course “Health Care for People Deprived of Freedom” lies not only in the massive education of health professionals but also in the improvement of the health of this social group and its impact on society. This factor effectively contributes to the achievement of the SDGs by 2030.

The scientific findings we present were elicited through secondary data analysis. This methodological limitation could be addressed by further research using primary data collected through instruments to analyze health impacts on the prison system. Another crucial need is to expand further the massive education the course provides nationwide, which can be achieved through a communication plan.

The course “Health Care of Persons Deprived of Freedom” has reached a substantial number of participants across Brazil. This fact may characterize a real need for health professionals to gain knowledge to develop skills and abilities in prison health. Therefore, it is recommended that health authorities expand access to training for health professionals, besides creating a specific learning pathway for prison health that covers the triad: Criminal Police, Imprisoned People, and Health Professionals. A prison system-specific pathway may better qualify all actors involved in implementing prison health policy.

## Data availability statement

The datasets presented in this study can be found in online repositories. The names of the repository/repositories and accession number(s) can be found at: https://zenodo.org/record/6499752#.YnGOR_PML0r.

## Author contributions

JV, SD-T, EO, JM, and RV contributed to conception and design of the study. FF and PM organized the database and repository. RV and FF performed the analysis. JV, RV, FF, and MR wrote the first draft and sections of the manuscript. All authors contributed to manuscript revision, read, and approved the submitted version.

## Funding

The Norte-Grandense Foundation for Research and Culture and the Federal University of Rio Grande do Norte were responsible for financing the development of this work through the Decentralized Section Term (TED), signed by the Federal University of Rio Grande do Norte and the Ministry of Health of Brazil.

## Conflict of interest

The authors declare that the research was conducted in the absence of any commercial or financial relationships that could be construed as a potential conflict of interest.

## Publisher's note

All claims expressed in this article are solely those of the authors and do not necessarily represent those of their affiliated organizations, or those of the publisher, the editors and the reviewers. Any product that may be evaluated in this article, or claim that may be made by its manufacturer, is not guaranteed or endorsed by the publisher.
